# Reelin induces a radial glial phenotype in human neural progenitor cells by activation of Notch-1

**DOI:** 10.1186/1471-213X-8-69

**Published:** 2008-07-01

**Authors:** Serene Keilani, Kiminobu Sugaya

**Affiliations:** 1 Biomolecular Science Center, Burnett School of Biomedical Sciences, College of Medicine, University of Central Florida, 4000 Central Florida Blvd, Orlando, FL 32816-2364, USA

## Abstract

**Background:**

Reelin and Notch-1 signaling pathways have been recently found to be necessary to induce the expression of brain lipid binding protein (BLBP) and to promote the process extension and the maturation of the neuronal progenitors, the radial glial cells. In this study, we report the cross talk between these two pathways.

**Results:**

Both *in vitro *Reelin treatment and overexpression of Notch-1 intracellular domain (NICD) induced BLBP expression and a radial glial phenotype in an immortalized human neural progenitor (HNP) cell line, isolated from the cortex of 14 weeks old fetus. Reelin treatment increased the level of NICD, indicating that Reelin signaling directly activates Notch-1. In addition, reducing NICD release, by inhibiting γ-secretase activity, inhibited the Reelin-induced radial glial phenotype in human neural progenitor cells. Furthermore, we found that Dab-1, an adaptor protein downstream of Reelin, was co-immunoprecipitated with Notch-1 and NICD.

**Conclusion:**

These data indicate that Reelin signaling induces BLBP expression and a radial glial phenotype in human neural progenitor cells via the activation of Notch-1. This study suggest that Reelin signaling may act to fine tune Notch-1 activation to favor the induction of a radial glial phenotype prenataly and would thus offer an insight into how Notch-1 signaling leads to different cellular fates at different developmental stages.

## Background

Radial glial cells have been recently demonstrated to be the progenitors for the majority of the central nervous system (CNS) neurons [1,2]. They arise early in the development of the CNS, from the neuroepithelial cells lining the ventricles, around the time neurons start to appear [3]. Radial glial cells extend a long radial process to the pial surface while having their cell bodies in the ventricular zone (VZ), and thus allowing the migration of neurons from the VZ to the postmitotic areas [4]. However, the finding that these cells can also give birth to the migratory neurons via their asymmetric division [5,6] further highlighted the significance of these cells in brain development. Interestingly, radial glial cells disappear or transform into astrocytes in most regions of the mammalian brain after neuronal generation and migration are completed [3].

Brain lipid binding protein (BLBP) is a nervous system-specific member of the large family of hydrophobic ligand binding proteins and is exclusively expressed in radial glia and astrocytes during development throughout the CNS [7]. A recent elegant study using Cre/loxP fate mapping, showed that all neuronal population in the mouse brain are derived from radial glial cells expressing BLBP [2].

Recently, BLBP expression in cortical radial glia as well as its role in radial process extension was found to be dependent on Reelin expression [8]. Reelin is a large secreted glycoprotein, which has been shown to be an important signal for neuronal migration and proper positioning of neurons in the cerebral cortex and cerebellum [9]. Reelin signal is transmitted into the cells by Disabled-1 (Dab-1), a cytoplasmic adaptor protein that binds the internalization (NPXY) motif of the Reelin receptors, apolipoprotein receptor2 (ApoER2) and the very low density lipoprotein receptor (VLDLR), via its phospho-tyrosine binding (PTB) domain [10]. Tyrosine phosphorylation of Dab-1 is initiated after Reelin binding to its receptors [11], forming new binding sites for Src kinases [12,13]. Mice deficient in, VLDLR and ApoER2, Dab-1 or Dab-1 that cannot be tyrosine phosphorylated generates a reeler-like phenotype, which is characterized by inversion of the cortical layers and abnormalities in the laminated brain structures [14].

Reelin effect on BLBP expression requires Dab-1 as well, as it failed to occur in Dab-1-/- mice [8]. Reeler and Dab-1 mutant mice were shown to exhibit abnormal development of the radial glia scaffold in the dentate gyrus of the hippocampus [15]. Furthermore, components of the Reelin signaling pathway, including VLDLR, ApoER2, and Dab1 were shown to be expressed in both the hippocampul and cortical radial glia [15,16]. Interestingly, Reelin addition *in-vitro *has been shown not only to promote the process extension of the radial glial cells but also to rescue the defects in the length of these processes in the reeler mutant radial glia [8].

A recent study showed that Notch-1 signaling could also regulate the molecular and the morphological differentiation of radial glia through the transcriptional activation of brain lipid binding protein (BLBP) [17]. Notch is a single-pass transmembrane protein that was initially identified in Drosophila and has been shown to mediate many developmental signaling events [18]. Notch-1 activation, upon binding its ligand, will initiate its cleavage by γ-secretase to generate Notch-1 intracellular domain (NICD). NICD translocates to the nucleus and binds C-promoter Binding Factor 1 (CBF1), converting it from transcriptional repressor to transcriptional activator [19]. Interestingly, BLBP expression was found to be dependent on this pathway as well [17,20], where a binding site for the Notch-1 effectors' CBF1 was found in the promoter region of BLBP [21].

Since Notch-1 activation can induce both the radial glial phenotype and their transformation into astrocytes [22,23], we hypothesized that Reelin signaling may act to fine-tune Notch-1 activation favoring the induction of the radial glial phenotype prenataly. This in turn can explain how the degeneration of the cells that produces Reelin in the cortex coincides with transformation of the radial glial cells into astrocytes postnataly [24]. In our study, *in vitro *addition of Reelin to human neural progenitor cells (HNPCs), isolated from the cortex of 14 weeks old fetus, induced BLBP expression and a radial glial phenotype similar to that induced by Notch-1 activation. The inhibition of Notch-1 activation by inhibiting the activity of γ-secretase abolished Reelin's effect, suggesting the dependency of Reelin signaling on Notch-1 activation. Furthermore, Reelin treatment increased the level of NICD, indicating that Reelin can directly activate Notch-1. Finally, Dab-1 was observed to bind to Notch-1, thus providing an evidence of the physical interaction between these two pathways.

## Results

### Reelin treatment *in vitro *induces a radial glial phenotype similar to Notch-1 activation

Reelin addition *in vitro *has been recently shown to promote the process extension of the radial glial cells and to rescue the defects in the length of these processes in the reeler mutant radial glia [8]. To test if Reelin signaling acts on Notch-1 activation to promote the radial glial phenotype, we first compared the induced radial glial phenotype obtained after Reelin treatment and Notch-1 activation. NICD overexpression was used to mimic Notch-1 activation.

**Figure 1:**
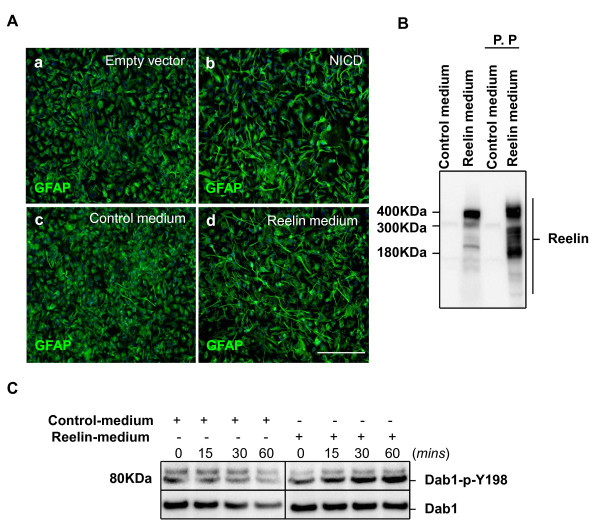
**Reelin treatment *in vitro *induces a radial glial phenotype similar to Notch-1 activation**. ***A***, HNPCs were deprived of FGF and EGF for 24 hours prior to their transfection or treatment. ***a***, HNPCs were transfected with empty vector or ***b ***NICD for 24 hours before fixation. ***c***, HNPCs were treated with control or ***d***, partially purified Reelin for 24 hours before fixation. The cells were later stained against GFAP. Scale bar 200 μm. ***B***, Control and Reelin conditioned-medium were collected from HEK293 cells transfected with either control empty vector or Reelin, respectively. Reelin was partially purified (P.P) from the medium by column centrifugation and its expression was verified by western blotting using anti-reelin clone G10. ***C***, Reelin treatment of HNPCs *in vitro *(1:40) induced Reelin signaling by increasing Dab-1 phosphorylation on tyrosine residue 198.

It has been reported that FGF and EGF signaling can also promote the radial glial phenotype [29,30]. In order to characterize only the effects of Reelin treatment or Notch-1 activation on HNPCs, we deprived the cells from these growth factors for 24 hours before Reelin addition or NICD over expression (Figure 1A). The deprivation of these growth factors reduced the radial glial phenotype, which was characterized by the retraction of the processes in the GFAP positive cells (data not shown). The cells were then treated with partially purified Reelin (Figure 1B) or transfected with NICD for 24 hours before fixation.

As a result, Reelin treatment was observed to induce a radial glial phenotype, which was characterized by increased process extension in the GFAP positive cells (Figure 1A.d), similar to that obtained by Notch-1 activation (Figure 1A.b).

To confirm that Reelin treatment activates Reelin signaling in HNPCs, we measured the phosphorylation level of the tyrosine residue 198 of Dab-1 after treating the cells with Reelin for 15 minutes, 30 minutes and 1 hour. Dab-1 phosphorylation increased after Reelin treatment, whereas it remained constant with the control treatment (Figure 1C), indicating that these cells are responsive to Reelin.

### Reelin-induced radial glial phenotype is dependent on γ-secretase activity

**Figure 2:**
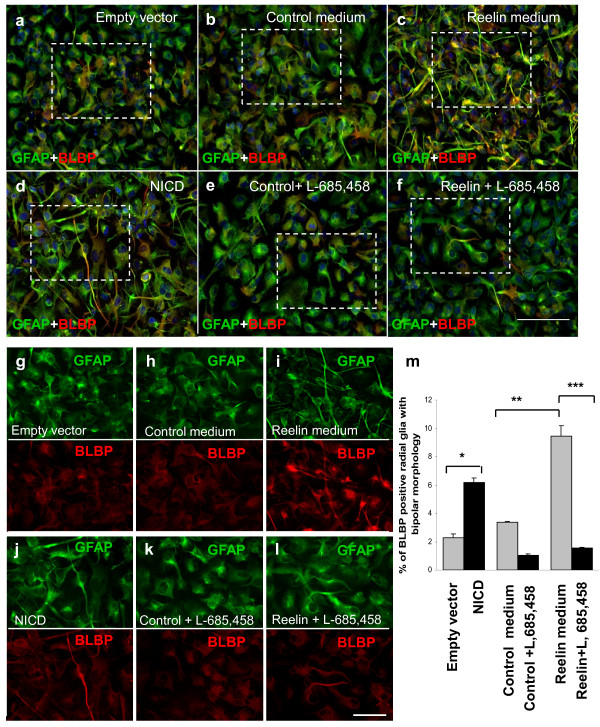
**Reelin-induced radial glial phenotype is dependent on γ-secretase activity**. HNPCs were deprived of FGF and EGF for 24 hours prior to their transfection or treatment. ***a, g***, HNPCs were transfected with empty vector or ***d, j ***NICD for 24 hours before fixation. The percentage of the BLBP-positive radial glia increased from 2.3% in the empty vector transfected cells to 6.2% in the NICD transfected cells, *p-value = 0.05. ***b, h, ***HNPCs were treated with partially purified control or ***c, i, ***partially purified Reelin for 24 hours before fixation. The percentage of the BLBP-positive radial glia increased from 3.4 % in the control-treated cells to 9.5% in the Reelin-treated cells, **p-value = 0.05. ***e, k, f, l***, 10 μM of L-685,458 was used to inhibit the activity of γ-secretase for 24 hours in the control and the Reelin treated HNPCs. γ-secretase inhibition in the Reelin treated cells abolished Reelin's effect by reducing the percentage of the BLBP-positive radial glia back to 1.6 %, ***p-value = 0.05. The results represent the standard error of the mean ± SEM of at least three independent experiments. One-way ANOVA with Tukey's post hoc test for individual treatment differences was used for statistical analysis. Values that are significantly different from each other according to Tukey's test are indicated by asterisks. Scale bar for a-f is 100 μm and for g-l is 50 μm.

To test the dependency of Reelin signaling on Notch-1 activation to induce the radial glial phenotype, we blocked NICD release by inhibiting γ-secretase activity. The cells were deprived of FGF and EGF for 24 hours and then treated with partially purified Reelin and 10 μM of the γ-secretase inhibitor (L- 685, 458) for another 24 hours before analysis (Figure 2).

As expected, Reelin treatment and NICD overexpression up-regulated BLBP expression and induced a bipolar radial morphology (Figure 2c,d,i and 2j). The induced radial glial phenotype was determined by finding the percentage of the GFAP positive cells that had induced BLBP expression and a bipolar morphology with at least one thin process longer than 50 μm.

As a result, we found that the percentage of the radial glial cells increased from 3.4 % in the control-treated cells to 9.5% in the Reelin-treated cells (at **p-value = 0.05, q_*observed *_(26.403)> q_*critical *_(4.751) by *post-hoc *Tukey *t *test) (Figure 2m). The inhibition of γ-secretase activity in the Reelin-treated cells reduced the percentage of the radial glial cells back to 1.6 % (at ***p-value= 0.05, q_*observed *_(24.744)> q_*critical *_(4.751) by *post-hoc *Tukey *t *test) (Figure 2m). These data suggest the dependency of Reelin on the activity of γ-secretase to induce the radial glial phenotype in HNPCs.

### Reelin treatment activates Notch-1

**Figure 3:**
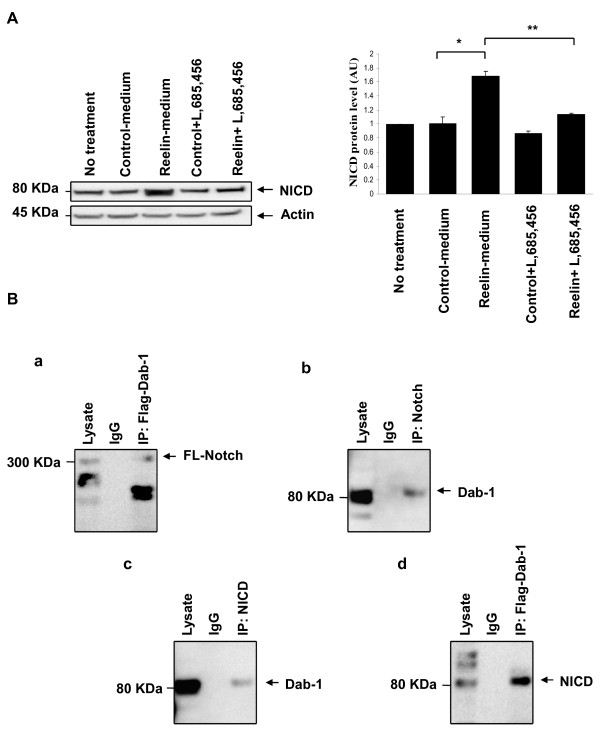
**Reelin treatment activates Notch-1**. ***A***, HNPCs were treated with partially purified control or Reelin with and without γ-secretase inhibition for 24 hours before analysis. Reelin addition *in vitro *increased the accumulation of NICD by 1.7 fold in the treated HNPCs, * p-value = 0.05, where γ-secretase inhibition abolished this increase, ** p-value = 0.05. The results represent the standard error of the mean ± SEM of at least three independent experiments. One-way ANOVA with Tukey's post hoc test for individual treatment differences was used for statistical analysis. Values that are significantly different from each other according to Tukey's test are indicated by asterisks ***B***, Western blots showing reciprocal co-immunoprecipitation (IP). HNPCs were transfected with Dab-1 with a flag tag added to its C terminus for 24 hours before cell lysis. ***a***, Co-immunoprecipitation of Notch-1 by Dab-1. ***b***, Co-immunoprecipitation of Dab-1 by Notch-1. ***c***, Co-immunoprecipitation of Dab-1 by NICD. ***d***, Co-immunoprecipitation of NICD by Dab-1. Anti-Flag was used to immunoprecipitate Dab-1 from the cell lysate.

To determine if Reelin affect Notch-1 signaling, we measured the protein level of NICD by western blotting using an antibody that can recognize only the activated form of Notch-1 (NICD). As a result, Reelin treatment significantly increased NICD level by 1.7 fold when compared to control treatment (at *p-value= 0.05, q_*observed *_(11.601)> q_*critical*_(4.654) by *post-hoc *Tukey *t *test). In contrast, the inhibition of γ-secretase activity in the Reelin-treated cells reduced NICD level back to 1.1 fold (at **p-value= 0.05, q_*observed *_(9.327)> q_*critical *_(4.654) by *post-hoc *Tukey *t *test) (Figure 3A).

Since Notch-1 has the NPXY motif that is recognized by Dab-1's PTB domain [31], we tested if Dab-1 binds to Notch-1. The cells were transfected with flag tagged Dab-1 for 24 hours (Figure 3B). An immunoprecipitation using an antibody against the flag tag on the C-terminal of Dab-1 was able to co-immunoprecipitate full-length Notch-1 (Figure 3B.a). Similarly, an immunoprecipitation using an antibody recognizing Notch-1 co-immunprecipitated Dab-1 (Figure 3B.b). These results indicate that full-length Notch-1 and Dab-1 can interact.

We also investigated if Dab-1 can bind to the NICD. The cells were transfected with flag tagged Dab-1 for 24 hours. Dab-1 was detected when the protein samples were immunoprecipitated using an antibody that recognizes only NICD (Figure 3B.c). Similarly, when the protein samples were immunoprecipitated by antibody recognizing the flag tag on the C-terminal of Dab-1, NICD was detected (Figure 3B.d). These results indicate that Dab-1 does not only bind to full-length Notch1 but also to the NICD.

## Discussion and Conclusions

Reelin and Notch-1 signaling pathways have been recently found to be necessary to induce the expression of BLBP and to promote the process extension and the maturation of the neuronal progenitors, the radial glial cells [8,17,20,21].

The end of neurogenesis in the cortex is marked by the transformation of the radial glial cells into astrocytes [3]. Notch-1 activation has been known for its role in promoting both phenotypes, the formation of the radial glial cells and their transformation into astrocytes. Interestingly, Notch-1 activation has been shown to induce radial glial cells differentiation prenataly and to induce astrocytic differentiation postnataly [22,23]. Therefore, the presence of other signaling pathways that act to induce the radial glial phenotype prenataly but are deactivated or downregulated postnataly seems to be an attractive model to explain how Notch-1 activation leads to different cellular fates at different developmental stages.

Reelin signaling has been recently shown to induce the radial glial phenotype *in vitro *and to rescue the defects in the reeler mutant radial glia [8]. Interestingly, Cajal-Retzius cells, the cells that produce Reelin in the cortical marginal zone, are thought to either degenerate postnatally [24] or undergo developmental dilution [25].

Here, we propose that Reelin signaling promotes Notch-1 activation to favor the radial glial phenotype prenataly. To test our hypothesis, we used human neural progenitor cells that were isolated form the cortex of 14 weeks fetus. We show that these cells responded to Reelin treatment by inducing a bipolar morphology in the GFAP positive cell indicating the formation of radial glia (Figure 2c). Since this Reelin-induced phenotype was dependent on γ-secretase activity (Figure 2f), and since Reelin treatment increased the level of NICD (Figure 3A), then we concluded that Reelin signaling promote the radial glial phenotype by activating Notch-1.

Reelin signaling might regulate Notch-1 activation via Dab-1 binding to Notch-1 (Figure 3B). It's possible that Reelin signaling mediated by Dab-1, increases the NICD level by either increasing Notch-1 processing by γ-secretase or by stabilizing the released NICD. Since Dab-1 has been recently shown to act as a nucleocytoplasmic shuttling protein [32], Dab-1 may in turn mediate the translocation of NICD into the nucleus to activate downstream genes such as BLBP. However, the exact mechanism needs further elucidation.

Taken together, this study offers a new insight of how Reelin and Notch-1 signaling are determining that fate of the neural progenitor cells and maintaining the radial glial phenotype in the developing brain.

## Methods

### Cell Culture

We used ReNcell CX (Chemicon), an immortalized human neural progenitor cell line (HNPCs) with the ability to readily differentiate into neurons and glial cells. ReNcell CX was derived from the cortical region of a 14-weeks human fetal brain. Immortalized by retroviral transduction with the c-myc oncogene. The cells were cultured in ReNcell NSC maintenance media (Chemicon) supplemented with 20 ng/mL of the fibroblast growth factor (FGF) and 20 ng/mL and of the epidermal growth factor (EGF) (Chemicon).

The cells were deprived of the growth factors, FGF and EGF, for 24 hours before Reelin treatment or NICD overexpression to exclude the effects of these factors on the induced radial glial phenotype.

For transfection of HNPCs, the cells were plated on laminin coated (Sigma Aldrich) 12-well culture plates (BD biosciences) for 24 hours or until they reached 90% confluency. The cells were then transiently transfected with 2-μg of DNA/well using lipofectamine LTX and Plus reagents according to manufacture's instructions (Invitrogen). The transfection efficiency was determined by finding the percentage of the GFP fluorescent cells 24 hours after transfecting the HNPCs with EGFP vector (Invitrogen). The transfection efficiency was optimized by transfecting the cells with different ratios of DNA/PLUS/LTX, where the best transfection efficiency was found to be 30–40%, using the ratio of 1/3/3, respectively.

For γ-secretase inhibition, 10 μM of γ-secretase inhibitor (L-685, 458; Sigma Aldrich) or 1% of Dimethysulfoxide (DMSO; Sigma Aldrich) as control, were prepared in growth media and added to the cells for 24 hours before analysis.

Dab-1 gene (Accession # NM_021080) was cloned into pcDNA3.1 vector with FLAG tag on its C terminus (Invitrogen); sequencing was performed to assure that the clone is in frame. FCDN1 expressed in pBOS-EF1 (1747–2531 aa) was kindly provided by Dr. Gerry weinmaster, UCLA.

### Reelin production and treatment

Reelin- and control-conditioned media was collected from HEK293 cells stably transfected with Relin pCRL vector (kindly provided by T. Curran, St. Jude Children's Hospital, Memphis Tennessee, USA), and a control empty pcDNA3.1 vector (Invitrogen), respectively [15,26,27]. The cells were cultured in neurobasal media (Invitrogen) for 3 days before media collection. Reelin was partially purified using columns with filters >100 kDa (Chemicon) and Reelin level was confirmed by western blotting using anti-Reelin clone G10 (Chemicon) that detected the three characteristic bands of Reelin (400 KDa, 300 KDa, and 180 KDa) [11].

HNPCs were treated with either partially purified control or Reelin at a final concentration of 1:40 diluted in ReNcell CX maintenance media for 24 hours. The concentration of partially purified Reelin in the conditioned media was determined by finding the lowest concentration of Reelin that can induce Dab-1 phosphorylation as early as 15 minutes (data not shown).

### Protein Isolation and Western Blot Analysis

HNPCs were lysed in ice-cold RIPA (Radio-Immunoprecipitation Assay) lysis buffer (Sigma Aldrich) containing 150 mM NaCl, 1% IGEPAL CA-630, 0.5% sodium deoxycholate, 0.1% SDS, and 50 mM Tris, pH 8.0, supplemented with 1% Triton-X and protease inhibitor mixture (Calbiochem). The homogenates were centrifuged at 12,000 × *g *for 30 min at 4°C and the supernatants were saved for analysis. Forty micrograms of protein was loaded per well, and proteins were separated by SDS/PAGE (NuPage 4%–12% Bis-Tris, Invitrogen) and then blotted onto polyvinyldiene difluride (PVDF) membranes (Bio-rad) for 120 min at 30 V. For the detection of full-length Notch-1, activated Notch-1, Reelin, BLBP, β-actin, Dab-1 and p198Dab-1, membranes were incubated overnight with following primary antibodies, respectively: mouse anti-Notch-1 (1:1,000; Sigma Aldrich), rabbit anti-activated Notch-1 (1:500; Abcam), mouse anti-Reelin clone G10 (1:1000; Chemicon), rabbit anti-BLBP (1:500; Abcam), rabbit anti-β-actin (1:1,000; Cell Signaling Technology, Danvers, MA), rabbit anti-Dab-1 (1:1000;Sigma Aldrich) and rabbit anti-Dab-1 phospho tyrosine 198 (1:1000; Chemicon). After washing, membranes were incubated with horseradish peroxidase-conjugated secondary antibodies at concentration of 1:5000 (anti-mouse IgG and anti-rabbit IgG; Jackson Immunoresearch, West Grove, PA) for 1–2 h. Signals were detected by chemiluminescence using the enhanced chemiluminescence (ECL) system (Amersham Biosciences Corp) on a Kodak Imaging Station IS2000 MM. The optical density of each specific band relative to β-actin was analyzed by the public domain National Institutes of Health Image J software.

### Immunoprecipitation

700 μg of protein was incubated for 3 hours at 4°C with the primary antibody anti-Notch-1 (1:100; Sigma Aldrich), or anti-Flag (1:200; Sigma Aldrich), or anti-NICD (1:500; Abcam), Protein G- or A-Sepharose beads were added for 1 hr at 4°C, and then immunoprecipitates were washed 5× with lysis buffer. Immunoprecipitated proteins were solubilized in 1% SDS solution and resolved on a 4–12% Bis-Tris gel (Invitrogen).

### Fluorescent Immunocytochemistry

HNPCs were fixed with 4% Paraformylaldehyde (Sigma Aldrich) overnight at 4°C. The next day, the cells were washed three times with phosphate buffered saline (PBS- Sigma Aldrich) at room temperature and blocked with 3% donkey serum (Jackson ImmunoResearch Labs) in phosphate buffered saline with 0.1% Triton-X (PBST- Sigma Aldrich) for 1 hour at room temperature. For analyzing the radial glial phenotypes, HNPCs were incubated with rabbit anti-BLBP (1:50; Abcam) and mouse anti- GFAP (1:500; Sigma Aldrich) for 24 hrs at 4°C. After washing the cells with PBS for three times the cells were incubated with rhodamine TRTIC-conjugated anti-rabbit and fluorescein FITC-conjugated anti-mouse (1: 500; Jackson ImmunoResearch Labs) for 2 hrs at room temperature in the dark. The cells were washed again and mounted with vectasheild with DAPI (vector laboratories) and cover slipped for microscopic observations.

### Microscopy and analysis of the radial glial phenotype

The induced radial glial phenotype was calculated by finding the percentage of the GFAP positive cells that had an induced BLBP expression [33] and a bipolar morphology, with at least one thin process longer than 50 μm. This criterion was based on previous studies [8,28]. Adobe Photoshop CS3 software was used to count the number of cells. Microscopic images were taken with an Axiocam digital camera (Zeiss, Oberkochen, Germany) mounted on the DMRB and processed using the QI imaging with Q capture software (Q Imaging, Burnaby, Canada). A total of three separate trials were performed. In each trial three random fields were selected for each condition. A total of ~600 cells were counted in each field (10× magnification).

### Statistical Analysis

Each data point represents triplicated experiments and displayed as the mean ± standard error. A one-way Analysis of Variance was performed to test the hypothesis that the average mean values across categories of GROUP were equal. In the presence of significance for the omnibus ANOVA test, Tukey multiple comparison test is used to perform pairwise comparisons. Statistics were performed using WINKS SDA Software (Texasoft, Cedar Hill, TX.). For immunocytochemistry and cell count: The average values across categories of GROUP were found to be different. F (5, 12) = 103.22, p < .001. For Western: The average values across categories of GROUP were found to be different. F (4, 10) = 29.84, p < .001. A Tukey multiple comparison procedure was performed at p = 0.05.

## Authors' contributions

All authors read and approved the final manuscript.

## References

[B1] Noctor SC, Flint AC, Weissman TA, Wong WS, Clinton BK, Kriegstein AR (2002). Dividing precursor cells of the embryonic cortical ventricular zone have morphological and molecular characteristics of radial glia. J Neurosci.

[B2] Anthony TE, Klein C, Fishell G, Heintz N (2004). Radial glia serve as neural progenitors in all regions of the central nervous system. Neuron.

[B3] Kriegstein AR, Gotz M (2003). Radial glia diversity: a matter of cell fate. GLIA.

[B4] Gaiano N, Fishell G (2002). The role of Notch in promoting glial neural stem cell fates. Annu Rev Neurosci.

[B5] Noctor SC, Flint AC, Weissman TA, Dammerman RS, Kriegstein AR (2001). Neurons derived from radial glial cells establish radial units in neocortex. Nature.

[B6] Fishell G, Kriegstein AR (2003). Neurons from radial glia: the consequences of asymmetric division. Curr opin Neurobiol.

[B7] Hartfuss E, Galli R, Heins N, Gotz M (2001). Characterization of CNS precursor subtypes and radial glia. Dev Biol.

[B8] Hartfuss E, Forster E, Bock HH, Hack MA, Leprince P, Luque JM, Herz J, Frotscher M, Götz M (2003). Reelin signaling directly affects radial glia morphology and biochemical maturation. Development.

[B9] D'Arcangelo G, Miao GG, Chen SC, Scares HD, Morgan JI, Curran T (1995). A protein related to extracellular matrix proteins deleted in the mouse mutant reeler. Nature.

[B10] Forster E, Jossin Y, Zhao S, Chai X, Frotscher M, Goffinet AM (2006). Recent progress in understanding the role of Reelin in radial neuronal migration, with specific emphasis on the dentate gyrus. Eur J Neurosci.

[B11] Benhayon D, Magdaleno S, Curran T (2003). Binding of purified Reelin to ApoER2 and VLDLR mediates tyrosine phosphorylation of Disabled-1. Brain Res Mol Brain Res.

[B12] Bock HH, Herz J (2003). Reelin Activates Src Family Tyrosine Kinases in Neurons. Curr Biol.

[B13] Ballif BA, Arnaud L, Cooper JA (2003). Tyrosine phosphorylation of Disabled-1 is essential for Reelin-stimulated activation of Akt and Src family kinases. Brain Res Mol Brain Res.

[B14] Trommsdorff M, Gotthardt M, Hiesberger T, Shelton J, Stockinger W, Nimpf J, Hammer RE, Richardson JA, Herz J (1999). Reeler/Disabled-like disruption of neuronal migration in knockout mice lacking the VLDL receptor and ApoE receptor 2. Cell.

[B15] Forster E, Tielsch A, Saum B, Weiss KH, Johanssen C, Graus-Porta D, Müller U, Frotscher M (2002). Reelin, Disabled 1, and beta 1 integrins are required for the formation of the radial glial scaffold in the hippocampus. Proc Natl Acad Sci USA.

[B16] Luque JM, Morante-Oria J, Faire'n A (2003). Localization of ApoER2, VLDLR and Dab1 in radial glia: groundwork for a new model of reelin action during cortical development. Brain Res Dev Brain Res.

[B17] Patten BA, Peyrin JM, Weinmaster G, Corfas G (2003). Sequential signaling through *Notch1 *and erbB receptors mediates radial glia differentiation. J Neurosci.

[B18] Parks AL, Huppert SS, Muskavitch MA (1997). The dynamics of neurogenic signaling underlying bristle development in Drosophila melanogaster. Mech Dev.

[B19] Lai EC (2002). Keeping a good pathway down: transcriptional repression of Notch pathway target genes by CSL proteins. EMBO Rep.

[B20] Patten BA, Sardi SP, Koirala S, Nakafuku M, Corfas G (2006). Notch1 signaling regulates radial glia differentiation through multiple transcriptional mechanisms. J Neurosci.

[B21] Anthony TE, Mason HA, Gridley T, Fishell G, Heintz N (2005). Brain lipid-binding protein is a direct target of Notch signaling in radial glial cells. Genes Dev.

[B22] Gaiano N, Nye JS, Fishell G (2000). Radial glial identity is promoted by Notch1 signaling in the murine forebrain. Neuron.

[B23] Chambers CB, Peng Y, Nguyen H, Gaiano N, Fishell G, Nye JS (2001). Spatiotemporal selectivity of response to Notch1 signals in mammalian forebrain precursors. Development.

[B24] Super H, Uylings HB (2001). The early differentiation of the neocortex: a hypothesis on neocortical evolution. Cereb Cortex.

[B25] Martin R, Gutierrez A, Penafiel A, Marin-Padilla M, de la Calle A (1999). Persistence of Cajal-Retzius cells in the adult human cerebral cortex. An immunohistochemical study. Histol Histopathol.

[B26] Beffert U, Morfini G, Bock HH, Reyna H, Brady ST, Herz J (2002). Reelin-mediated signaling locally regulates PKB/Akt and GSK-3β. J Biol Chem.

[B27] D'Arcangelo G, Nakajima K, Miyata T, Ogawa M, Mikoshiba K, Curran T (1997). Reelin is a secreted glycoprotein recognized by the CR-50 monoclonal antibody. J Neurosci.

[B28] Rio C, Rieff HI, Qi P, Khurana TS, Corfas G (1997). Neuregulin and erbB receptors play a critical role in neuronal migration. Neuron.

[B29] Gregg C, Weiss S (2003). Generation of functional radial glial cells by embryonic and adult forebrain neural stem cells. J Neurosci.

[B30] Yoon K, Nery S, Rutlin ML, Radtke F, Fishell G, Nicholas Gaiano N (2004). Fibroblast growth factor receptor signaling promotes radial glial identity and interacts with Notch signaling in telencephalic progenitors. J Neurosci.

[B31] Howell BW, Lanier LM, Frank R, Gertler FB, Cooper JA (1999). The disabled 1 phosphotyrosine-binding domain binds to the internalization signals of transmembrane glycoproteins and to phospholipids. Mol Cell Biol.

[B32] Honda T, Nakajima K (2006). Mouse Disabled1 (DAB1) is a nucleocytoplasmic shuttling protein. J Biol Chem.

[B33] Feng L, Heintz N (1995). Differentiating neurons activate transcription of the brain lipid binding protein gene in radial glia through a novel regulatory element. Development.

